# Prenylcysteine Oxidase 1 Is a Key Regulator of Adipogenesis

**DOI:** 10.3390/antiox12030542

**Published:** 2023-02-21

**Authors:** Cristina Banfi, Alice Mallia, Stefania Ghilardi, Maura Brioschi, Erica Gianazza, Sonia Eligini, Pelin Sahlén, Roberta Baetta

**Affiliations:** 1 Centro Cardiologico Monzino IRCCS, Unit of Functional Proteomics, Metabolomics, and Network Analysis, 20138 Milan, Italy; 2 Dipartimento di Biologia e Biotecnologie “Lazzaro Spallanzani”, Università di Pavia, 27100 Pavia, Italy; 3 Science for Life Laboratory, KTH—Royal Institute of Technology, Tomtebodavägen 23A, 17165 Solna, Sweden

**Keywords:** adipogenesis, prenylcysteine oxidase 1, adipose tissue, adipogenic factors, oxidation

## Abstract

The process of adipogenesis involves the differentiation of preadipocytes into mature adipocytes. Excessive adipogenesis promotes obesity, a condition that increasingly threatens global health and contributes to the rapid rise of obesity-related diseases. We have recently shown that prenylcysteine oxidase 1 (PCYOX1) is a regulator of atherosclerosis-disease mechanisms, which acts through mechanisms not exclusively related to its pro-oxidant activity. To address the role of PCYOX1 in the adipogenic process, we extended our previous observations confirming that Pcyox1^−/−^/Apoe^−/−^ mice fed a high-fat diet for 8 or 12 weeks showed significantly lower body weight, when compared to Pcyox1^+/+^/Apoe^−/−^ mice, due to an evident reduction in visceral adipose content. We herein assessed the role of PCYOX1 in adipogenesis. Here, we found that PCYOX1 is expressed in adipose tissue, and, independently from its pro-oxidant enzymatic activity, is critical for adipogenesis. *Pcyox1* gene silencing completely prevented the differentiation of 3T3-L1 preadipocytes, by acting as an upstream regulator of several key players, such as FABP4, PPARγ, C/EBPα. Proteomic analysis, performed by quantitative label-free mass spectrometry, further strengthened the role of PCYOX1 in adipogenesis by expanding the list of its downstream targets. Finally, the absence of *Pcyox1* reduces the inflammatory markers in adipose tissue. These findings render PCYOX1 a novel adipogenic factor with possible pathophysiological or therapeutic potential.

## 1. Introduction

Adipose tissue plays an important role in energy homeostasis through its capacity to store energy, mobilize stored lipids for fuel, and secrete hormones and cytokines [1]. Cardiovascular diseases, type 2 diabetes, and metabolic syndrome are all associated with excess fat accumulation in white adipose tissue (WAT) [2]. Understanding the molecular events that regulate adipocyte differentiation is an essential step towards preventing obesity and metabolic diseases because adipocyte differentiation determines adipose tissue mass.

Adipocyte differentiation involves hormonal stimulators and the induction of a network of transcription factors that induce changes in gene expression and cell morphology. At the top of this transcriptional hierarchy are the basic region/leucine zipper proteins CCAAT-enhancer binding protein (C/EBP)δ, C/EBPβ, C/EBPα, and the nuclear receptor peroxisome proliferator-activated receptor (PPAR)γ [3]. These transcription factors are induced in a sequential and tightly regulated manner, and all are essential for normal adipogenesis. However, several important unanswered questions remain, and a more complete understanding of the developmental origin as well as the cellular and molecular components of adipose tissue is necessary to further illuminate how excess adiposity contributes to the onset and/or progression of metabolic disorders.

We have recently shown that prenylcysteine oxidase 1 (PCYOX1) deficiency in apolipoprotein E deficient mice (Apoe^−/−^) is associated with reduced body weight and adipose tissue deposits [4], but the role of PCYOX1 in adipogenesis has never been addressed since its discovery in 1997 by Casey’s group in studies related to the metabolism of prenylated proteins [5,6]. 

To substantiate the contribution of PCYOX1 in the adipogenic process, we investigated its role in vitro and in vivo, taking advantage of a complementary approach based on gene silencing and label-free quantitative proteomics. 

Here we describe for the first time the role of PCYOX1 as a novel regulator of adipogenesis. Employing proteomics and gene expression analysis in *Pcyox1* silenced cells, we show that it acts upstream of the key factors that control the adipogenic process, while the in vivo absence of the *Pcyox1* gene in mice is associated with reduced adipose deposits, and related adipose tissue inflammation. Overall, these data identify PCYOX1 as a master regulator and potential therapeutic target in obesity-related diseases.

## 2. Materials and Methods

### 2.1. Cell Culture and Induction of Differentiation 

3T3-L1 preadipocytes (American Type Culture Collection, Manassas, VA, USA) were maintained in Dulbecco’s Modified Eagle Medium (DMEM) (Gibco; Thermo Fisher Scientific, Milan, Italy) with 4500 mg/L glucose supplemented with 10% fetal bovine serum (EuroClone, Milan, Italy). To induce differentiation, 2 days after reaching confluence (day 0), cells were exposed to a cocktail of 0.5 mmol/L 3-isobutyl-1-methyl-xanthine (Sigma-Aldrich, Milan, Italy), 0.25 µmol/L dexamethasone (Sigma-Aldrich, Milan, Italy) and 1 µg/mL insulin (Sigma-Aldrich, Milan, Italy) for 48 h, followed by exposure to insulin 1 µg/mL alone for additional 3 days. After this period, the medium was replaced every 2 days with DMEM containing 10% fetal bovine serum until the ninth day.

### 2.2. PCYOX1 Overexpression in CHO Cells 

CHO cells overexpressing PCYOX1 were generated as previously described [4]. Briefly, an empty pcDNA5/FRT vector and a custom pcDNA5/FRT:PCYOX1 (Invitrogen; Thermo Fisher Scientific, Milan, Italy) were transfected into Flp-In-CHO cells (R758-07, Invitrogen; Thermo Fisher Scientific, Milan, Italy) using a pOG44 expression vector (V6005-20, Invitrogen; Thermo Fisher Scientific, Milan, Italy). The selection of PCYOX1-expressing clones was performed according to the Flip-In System protocol (K6010-01, Invitrogen; Thermo Fisher Scientific, Milan, Italy).

### 2.3. Stable Transfection with Short Hairpin RNA (shRNA)

Stable gene silencing of *Pcyox1* and *Cebpb* in 3T3-L1 cells was achieved using shRNA plasmids (Santa Cruz Biotechnology, Dallas, TX, USA), a pool of 3 target-specific lentiviral vector plasmids each encoding 19–25 nt (plus hairpin) shRNAs designed to knock down *Pcyox1* and *Cebpb* gene expression. A shRNA plasmid encoding a scrambled shRNA sequence that does not induce the degradation of any cellular message was taken as negative control (control cells, shNEG). Cells at 50–70% confluency were transfected for 24 h with a multiplicity of infection (MOI) of 5 for *Pcyox1* shRNA and 7.5 for *Cebpb* shRNA lentiviral particles, and 5 µg/mL of Polybrene (Santa Cruz Biotechnology, Dallas, TX, USA) for each well of a 12 well/plate and cultured in complete medium containing 2 µg/mL puromycin to allow selection.

### 2.4. Oil Red O Staining

Cells were washed with phosphate-buffered saline (PBS) and fixed with formalin (4%) for 1 h. After washing with water, cells were treated with 60% isopropanol for 5 min, and then stained with Oil Red O working solution prepared according to the manufacturer’s instructions (Sigma-Aldrich, Milan, Italy). 

### 2.5. Real-Time Quantitative Reverse Transcriptase PCR (qRT-PCR)

Cells were harvested in the lysis buffer, and RNA was extracted with the Total RNA Purification Kit (Norgen Biotek Corp., Thorold, ON, Canada). Adipose tissues were homogenized by means of TissueLyser II (QIAGEN, Milan, Italy) before RNA extraction with the Fatty Tissue RNA Purification Kit (Norgen Biotek Corp., Thorold, Ontario, Canada) and reverse transcribed (1 μg) as described [7]. The quality of cellular and tissue RNA was checked by the Agilent 2100 Bioanalyzer system (Agilent Technologies, Santa Clara, CA, USA). Real-time qRT-PCR was performed in triplicate with 2.5 μL of cDNA incubated in 22.5 μL IQ Supermix containing primers and SYBRGreen fluorescence dye (Bio-Rad Laboratories, Milan, Italy) using the iCycler Optical System (Bio-Rad Laboratories, Milan, Italy) with an initial denaturation for 3.3 min at 95 °C, followed by 50 cycles of amplification (15 s at 95 °C and 60 °C for 1 min), and by melting curve. The sequences of the primers used are listed in Supplementary Table S1. The other specific primers were purchased from QIAGEN (Milan, Italy): *Pcyox1*, *Pparg*, *Fabp4, Cebpa*, *Cebpb*, *Lipe*, *Car3*, *Plin1*, *Agpat2*, *Ces1f, Gpd1*, *Emr1*, *Itgam*, *Itgax*, *Lgals3*, and *Saa3,* and summarized in Table S2. Expression levels were calculated by Ct values normalized to the housekeeping *18s* rRNA or *Gapdh* genes using the 2^−ΔΔCT^ data analysis method. *Pcyox1* amplicons (120 bp) were analysed by electrophoresis in agarose gel 2% *w*/*v* containing GelRed (Biotium, Fremont, CA, USA) and visualized with Gel doc (Bio-Rad Laboratories, Milan, Italy).

### 2.6. Oxidized Low Density Lipoprotein (oxLDL) Assay 

The quantitation of oxLDL in the cell culture medium was performed by a quantitative immunoenzymatic assay according to the manufacturer’s instructions (MyBioSource, Inc., San Diego, CA, USA). Briefly, shNEG and shPcyox1 silenced cells were induced to differentiate for 9 days. After this period, medium was collected and oxLDL levels were measured. 

### 2.7. PCOYX1 Activity Assay 

PCYOX1 activity was assessed as previously described [4]. Briefly, H_2_O_2_ produced in cells by the PCYOX1 reaction was measured using the Amplex Red Kit (Life Technologies, Milan, Italy). In the presence of peroxidase, the Amplex Red reagent is converted by H_2_O_2_ into the red-fluorescent oxidation product resorufin. The resorufin produced was measured following the fluorescence emission in a microplate reader Infinite 200 (TECAN, Mannedorf, Switzerland), equipped for excitation at 530 nm and fluorescence emission detection at 590 nm. Results are expressed as picomoles of H_2_O_2_ /µg protein.

### 2.8. Label-Free Mass Spectrometry (LC-MS^E^) Analysis

Cell pellets were dissolved in 25 mmol/L NH_4_HCO_3_ containing 0.1% RapiGest (Waters Corporation, Milford, MA, USA), sonicated, and centrifuged at 13,000× *g* for 10 min. After 15 min of incubation at 80 °C, proteins were reduced with 5 mmol/L dithiothreitol (DTT) at 60 °C for 15 min, and carbamidomethylated with 10 mmol/L iodoacetamide for 30 min at room temperature in darkness. Digestion was performed with sequencing grade trypsin (Promega, Milan, Italy) (1 µg every 20 µg of proteins) overnight at 37 °C. After digestion, 2% trifluoroacetic acid (TFA) was added to hydrolyze RapiGest and inactivated trypsin. Tryptic peptides were used for label-free mass spectrometry analysis, LC-MS^E^, performed on a hybrid quadrupole-time of flight mass spectrometer (SYNAPT-XS, Waters Corporation, Milford, MA, USA) coupled with a UPLC Mclass system and equipped with a nano-source (Waters Corporation, Milford, MA, USA), as previously described [8,9]. Statistical analysis was performed by means of Progenesis QIP v 4.1 (Nonlinear Dynamics) using a Uniprot mouse protein sequence database (v2020). The mass spectrometry proteomics data have been deposited to the ProteomeXchange Consortium via the PRIDE [10] partner repository with the dataset identifier PXD039943 and 10.6019/PXD039943. 

### 2.9. GO Analysis

The Search Tool for the Retrieval of Interacting Genes/Proteins (STRING 10.5) database [11] was used to identify enriched Gene Ontology (GO) terms in the biological process, molecular function, or cellular component categories, as previously described [12]. The enrichment function of STRING, which calculates an enrichment *p* value based on a hypergeometric test using the method of Benjamini and Hochberg for correction of multiple testing (*p* value cut-off < 0.05), was used.

### 2.10. Mass Spectrometry-Based Quantification of PCYOX1

Tryptic peptides (0.5 μg/uL), obtained as described above, were added with the stable isotope labelled proteotipic PCYOX1 peptide (CPSIILHD(R) from Thermofisher Scientific (Milan, Italy), desalted with ZipTip C18 (Millipore, Burlington, MA, USA) according to the manufacturer’s instruction, and then dissolved in water with 0.1% formic acid before mass spectrometry analysis. Two microliters of each sample, containing 10 fmol/μL of labelled heavy peptide, were injected into a Xevo TQ-S micro triple quadrupole mass spectrometer coupled to a Waters ACQUITY ultra-performance liquid chromatography (UPLC) M-Class system through an ionKey source (Waters Corporation, Milford, MA, USA), and analysed as previously described [13]. 

### 2.11. Mice and Diets

Pcyox1^−/−^ mice were bred with Apoe^−/−^ mice (B6.129P2-Apoetm1Unc/J, stock 002052, JAX™ Mice Strain) as previously described [4]. Intercrosses of resulting Apoe^−/−^/Pcyox1^+/−^ mice generated offspring that entered the study. All procedures were approved by the Institutional Animal Care and Ethics Committee of the University of Milan and by the Ministry of Health DGSAF (N. 782-2020; approval 10 August 2020). Mice were housed in an air-conditioned room at 22 ±  0.5 °C with a 12-h lighting cycle and free access to food and water. For experiments, double knockout mice (Pcyox1^−/−^/Apoe^−/−^) and control mice (Pcyox1^+/+^/Apoe^−/−^) were fed ad libitum with a high fat diet (HFD) containing 0.2% cholesterol, 21.2% fat (42% kcal), and 17.5% protein by weight (Teklad diet TD.88137; Envigo, Milan, Italy), starting at 11 weeks of age and continuing for 8 or 12 weeks. After the indicated period of HFD, mice were anesthetized by intraperitoneal injection of ketamine hydrochloride (75 mg/kg) and medetomidine (1 mg/kg) prior to visceral fat harvest. Abdominal visceral adipose tissue (VAT) was rapidly removed, weighed, and snap frozen for RNA and mass spectrometry analyses. Each experimental session included animals of both genotypes (Pcyox^+/+^/Apoe^−/−^ *n* = 23; Pcyox^−/−^/Apoe^−/−^ *n* = 19), with animals being assigned to the experimental groups according to genotype, and with the investigators blinded to group assignment during all experimental stages and when evaluating outcome measures. 

### 2.12. Statistical Analysis

Data analysis was performed with GraphPad Prism 9.3 software (GraphPad Software Inc., San Diego, CA, USA). All data sets were tested for normality of distribution and analysed using the Student *t* test or analysis of variance (ANOVA) for multiple comparison followed by Dunnett’s or Tukey’s post hoc test as indicated. Statistical significance level was accepted at *p* < 0.05. 

## 3. Results

### 3.1. Pcyox1^−/−^ Mice Have Decreased Adiposity

**Figure 1:**
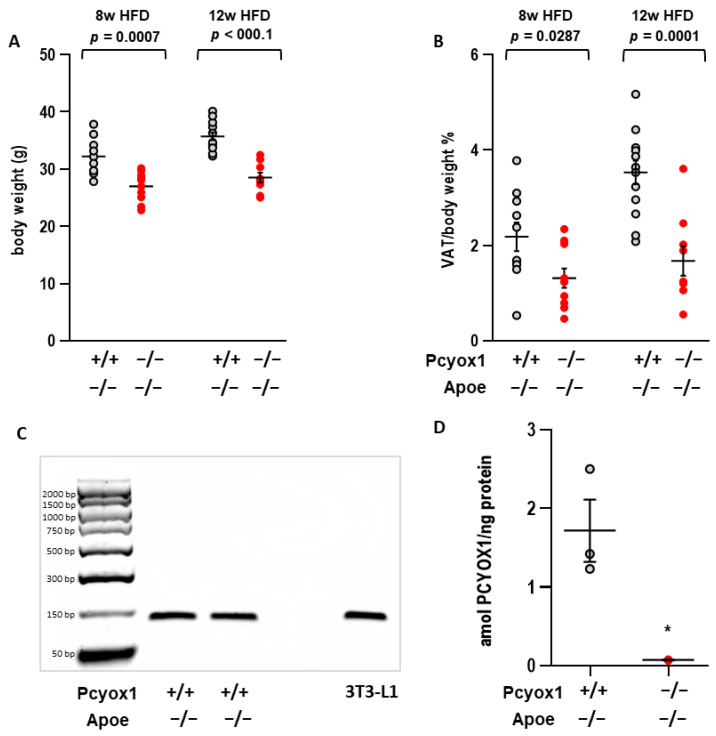
Effect of *Pcyox1* deficiency on (**A**) body weight and (**B**) visceral adipose tissue (VAT) content evaluated in male Pcyox1^+/+^/Apoe^−/−^ and Pcyox1^−/−^/Apoe^−/−^ mice fed an HFD for 8 weeks (Pcyox1^+/+^/Apoe^−/−^ *n* = 10; Pcyox1^−/−^/Apoe^−/−^ *n* = 10) and 12 weeks (Pcyox1^+/+^/Apoe^−/−^ *n* = 13; Pcyox1^−/−^/Apoe^−/−^ *n* = 9). (**C**) Representative gel image of *Pcyox1* mRNA expression, and (**D**) PCYOX1 protein level in VAT from wild-type mice evaluated by mass spectrometry (*n* = 3). Data are presented as circle plot, with each circle representing an individual mouse and bars showing the mean value  ±  SEM. VAT is expressed as percentage on body weight. HFD, high fat diet. Statistical significance calculated by Student’s *t* test, * *p* < 0.05 vs. Pcyox1^+/+^/Apoe^−/−^.

Pcyox1^−/−^/Apoe^−/−^ mice fed an HFD for 8 or 12 weeks showed 16.3% and 20.2% lower body weight, respectively, when compared to Pcyox1^+/+^/Apoe^−/−^ mice (Figure 1A), due to a 40% and 52.5% reduction in visceral adipose content (Figure 1B), thus confirming preliminary observations [4]. *Pcyox1* mRNA and protein, measured by quantitative mass spectrometry, were both present in the VAT of Pcyox1^+/+^/Apoe^−/−^ mice fed an HFD for 8 weeks (Figure 1C,D). As previously demonstrated, serum analysis in Pcyox1^−/−^/Apoe^−/−^ mice showed significantly decreased triglycerides, free fatty acid, and cholesterol concentrations, without any effects attributable to differences in food intake [4]. Overall, these data suggest that PCYOX1 may play a role in promoting in vivo adipogenesis. To address this, we explored the role of PCYOX1 in adipogenesis in in vitro differentiating cells.

### 3.2. PCYOX1 Expression Is Induced during Adipocyte Differentiation In Vitro

**Figure 2:**
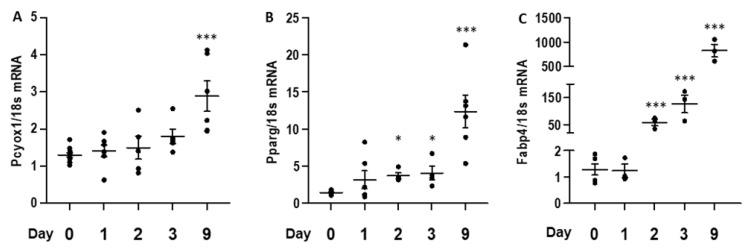
*Pcyox1* mRNA expression during 3T3-L1 in vitro differentiation. (**A**) *Pcyox1*, (**B**) *Pparg*, and (**C**) *Fabp4* mRNA levels during 3T3-L1 differentiation. *18s* mRNA was used as a housekeeping gene; *n* = 5–11 different experiments. * *p* < 0.05, *** *p* < 0.001 vs. day 0 with ANOVA and Dunnett’s post-hoc test.

*Pcyox1* mRNA expression is significantly increased when 3T3-L1 are fully differentiated to adipocyte-like cells after 9 days from the beginning of the differentiation protocol (Figure 2A). This occurs concomitantly with a rising expression of *Pparg*, a nuclear receptor critically involved in adipocyte differentiation, and *Fabp4*, a downstream target of PPARγ, which is a terminal marker of adipocyte differentiation (Figure 2B,C).

**Figure 3:**
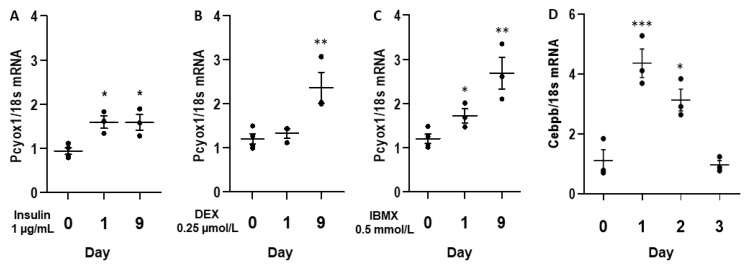
*Pcyox1* mRNA levels in 3T3-L1 during adipogenic differentiation in the presence of (**A**) insulin 1 µg/mL, (**B**), dexamethasone 0.25 µmol/L (DEX) and (**C**) the phosphodiesterase inhibitor 3-isobutyl-1-methylxanthine 0.5 mmol/L (IBMX). (**D**) mRNA levels of *Cebpb* during 3T3-L1 differentiation. *18s* mRNA was used as a housekeeping gene; *n* = 3–4 different experiments. * *p* < 0.05, ** *p* < 0.01, *** *p* < 0.001 vs. day 0 with ANOVA and Dunnett’s post-hoc test.

To explore the determinants of PCYOX1 induction during adipogenesis, we analysed the effects of the three components of the cocktail used to induce differentiation: insulin, dexamethasone (DEX), and the phosphodiesterase inhibitor 3-isobutyl-1-methylxanthine (IBMX). Insulin minimally perturbed *Pcyox1* expression (Figure 3A), whereas IBMX and dexamethasone strongly increased *Pcyox1* gene expression (Figure 3B,C). Because IBMX and DEX are each necessary for maximal and sustained expression of the pro-adipogenic transcription factor CCAAT/enhancer binding protein β (C/EBPβ) [14], we evaluate its mRNA modulation in the initial stages of differentiation (Figure 3D).

### 3.3. PCYOX1 Is Critical for Adipogenesis In Vitro

**Figure 4:**
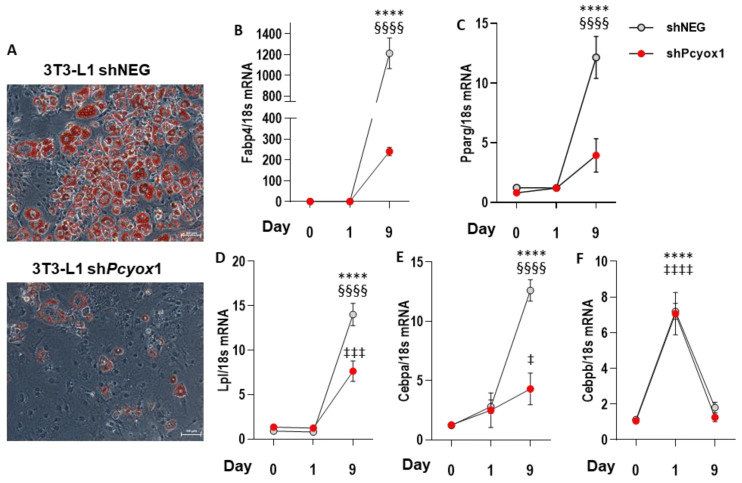
Effects of Pcyox1 silencing on 3T3-L1 differentiation towards adipocyte-like cells. Control cells were treated with lentiviral particles containing negative shRNA constructs (shNEG), while specific shPcyox1 constructs were used to silence *Pcyox1*. (**A**) Lipid droplets were visualized by Oil Red O staining after 9 days of differentiation. Representative images of *n*= 3 different experiments were acquired with 20× magnification. (**B**–**F**): effects of Pcyox1 silencing on mRNA levels of (**B**) *Fabp4*, (**C**) *Pparg*, (**D**) *Lpl*, (**E**) *Cebpa*, and (**F**) *Cebpb*. *18*s mRNA was used as a housekeeping gene; *n* = 3–7 different experiments. **** *p* < 0.0001 vs. shNEG day 0; §§§§ *p* < 0.0001 vs. shPcyox1 day 9; ‡‡‡‡ *p* < 0.0001 vs. shPcyox1 day 0; ‡‡‡ *p* < 0.001 vs. shPcyox1 day 0; ‡ *p* < 0.05 vs. shPcyox1 day 0 with ANOVA and Tukey’s post-hoc test.

To examine the role of PCYOX1 in adipogenesis, short-hairpin shRNA retroviral constructs targeting three regions of the *Pcyox1* mRNA were generated, and stably transfected into 3T3-L1 preadipocytes. The control and knockdown cell lines were then induced to differentiate for 9 days. Before the induction of differentiation, *Pcyox1* mRNA levels were reduced in silenced cells (−92.8 ± 1.1% with respect to control cells, *n* = 5, *p* < 0.001), and remained significantly reduced until day 9 (−86.4 ± 2.5%, *n* = 8, *p* < 0.001 versus control cells). Similarly, treatment of 3T3-L1 cells with the *Pcyox1* shRNA reduced endogenous PCYOX1 protein levels by 79.1 ± 6%, as assessed by mass spectrometry (*n* = 3; *p* < 0.0002). The shPcyox1-treated 3T3-L1 were also examined for their ability to differentiate into adipocytes compared to control cells. In response to adipogenic inducers, control cells underwent efficient morphological differentiation into lipid droplet-containing adipocytes. By contrast, *Pcyox1*-depleted 3T3-L1 preadipocytes accumulated fewer lipid droplets when induced to undergo differentiation (Figure 4A). 

The mRNA expression of *Fabp4*, a terminal marker of adipogenesis (Figure 4B), was found to be significantly reduced in *Pcyox1*-silenced cells compared to control cells at the end of differentiation.

To identify the developmental stage regulated by PCYOX1, we harvested control and *Pcyox1*-depleted 3T3-L1 cells at 0, 1, and 9 days after the addition of adipogenic inducers in order to evaluate the expression of known transcriptional factors involved in adipogenesis. *Pparg* (Figure 4C) and *Cebpa* (Figure 4D) were significantly lower in *Pcyox1*-silenced cells compared to controls at day 9. Importantly, the earlier induction of *Cebpb* was unaffected by the loss of PCYOX1 in 3T3-L1 cells, being induced to the same extent in control and *Pcyox1*-silenced cells 24 h after the addition of the adipogenic cocktail (Figure 4E), suggesting that PCYOX1 acts downstream of C/EBPβ to promote the adipogenic program.

**Figure 5:**
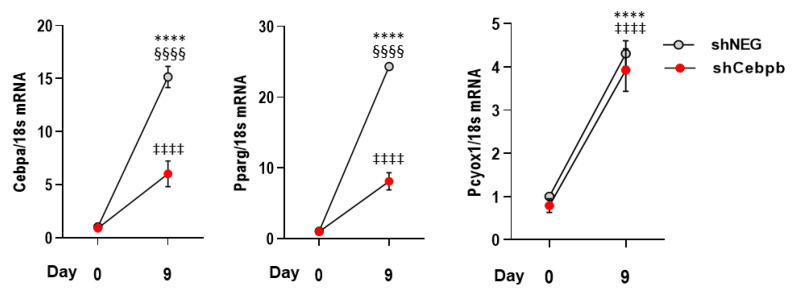
Effects of *Cebpb* silencing on *Cebpa*, *Pparg,* and *Pcyox1* expression during 3T3-L1 differentiation. *18s* mRNA was used as a housekeeping gene; *n* = 6 different experiments. **** *p* < 0.0001 vs. shNEG day 0; §§§§ *p* < 0.0001 vs. shCebpb; ‡‡‡‡ *p* < 0.0001 vs. shCebpb day 0 with ANOVA and Tukey’s post-hoc test.

To assess whether C/EBPβ can directly regulate PCYOX1, we silenced cells for *Cebpb*. Although there was a significantly lower expression of *Cebpb* (−54 ± 6% in silenced cells with respect to control cells treated with a negative construct, *n* = 3, *p* < 0.002) and the known C/EBPβ downstream targets *Pparg* and *Cebpa*, *Pcyox1* expression was unaffected (Figure 5).

### 3.4. The Effects of PCYOX1 in Adipogenesis Are Independent of Its Pro-Oxidant Activity

We previously demonstrated that PCYOX1 is able to generate H_2_O_2_, which in turn induces the formation of oxLDL [4], a known activator of PPARγ because a variety of oxLDL components act as PPARγ ligands [15]. Therefore, we assessed whether PCYOX1 could induce the formation of oxLDL during adipogenesis. First, we measured PCYOX1 activity in control and *Pcyox1*-silenced cells, in comparison with CHO cells overexpressing PCYOX1. The results obtained indicated that, in differentiated adipocytes, PCYOX1 is expressed but is not active (1.4 pmol H_2_O_2_/µg protein in CHO cells overexpressing PCYOX1, undetectable in differentiated adipocytes, *n* = 3). Furthermore, the levels of oxLDL, as assessed by immunoenzymatic assay, were not different between control cells and *Pcyox1*-silenced cells (1.1 ± 0.3 ng/mL and 1.0 ± 0.2 ng/mL, respectively, *n* = 6).

### 3.5. PCYOX1 Significantly Affects the Cell Proteome

The impact of PCYOX1 deletion on adipogenesis was further investigated by proteomics. Label-free quantitative mass spectrometry revealed that 43 proteins were significantly less abundant in the *Pcyox1*-deficient cells, and only two were more abundant in the *Pcyox1*-deficient cells compared to control cells (Table 1). 

**Figure 6:**
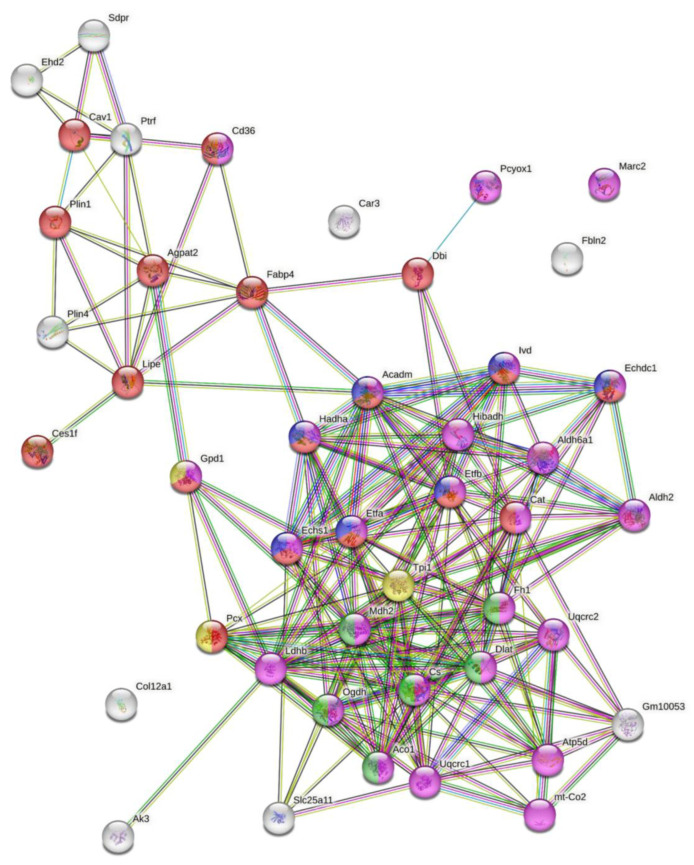
GO analysis of proteins modulated by *Pcyox1* silencing. Enriched GO terms in the biological process category are highlighted in different colors: red, lipid metabolic processes; violet, oxidation-reduction processes; blue, fatty acid β-oxidation; green, tricarboxylic acid cycle; yellow, gluconeogenesis.

The GO analysis of proteins that were less abundant after *Pcyox1* silencing (Figure 6 and Supplementary Table S3) revealed the enrichment of GO terms related to the lipid metabolic process (*n* = 18, *p* = 5.79e^−10^, i.e., hormone-sensitive lipase +(*Lipe*), fatty acid-binding protein (*Fabp4*), perilipin-1 (*Plin1*)), fatty acids β-oxidation (*n* = 7, *p* = 2.01e^−8^, i.e., enoyl-CoA hydratase), tricarboxylic acid cycle (*n* = 6, *p* = 4.21e^−8^, i.e., malate dehydrogenase 2, citrate synthase), gluconeogenesis (*n* = 3, *p* = 0.0069, i.e., glycerol-3-phosphate dehydrogenase), and oxidation reaction process (*n* = 26, *p* = 1.26e^−20^). 

**Figure 7:**
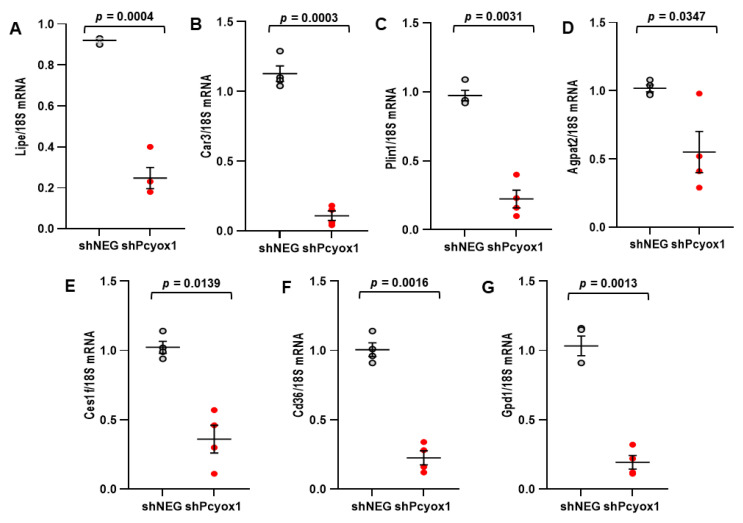
mRNA levels of the differently expressed proteins in *Pcyox1* silenced cells. mRNA levels were evaluated in control (shNEG) and *Pcyox1*-silenced (shPcyox1) 3T3-L1 cells after 9 days of adipocyte differentiation. (**A**) Hormone-sensitive lipase (*Lipe*), (**B**) Carbonic anhydrase 3 (*Car3*), (**C**) Perilipin-1 (*Plin1*), (**D**) 1-acyl-sn-glycerol-3-phosphate acyltransferase beta (*Agpat2*), (**E**) Carboxylesterase 1F (*Ces1f*), (**F**) Platelet glycoprotein 4 (*Cd36*) and (**G**) Glycerol-3-phosphate dehydrogenase [NAD(+)] cytoplasmic (*Gpd1*); *18s* mRNA was used as a housekeeping gene; *n* = 4 different experiments. Statistical significance calculated with Student’s *t* test.

Furthermore, the results from the proteomic analysis were validated in independent experiments by assessing the mRNA levels of the less abundant proteins: hormone-sensitive lipase (*Lipe*), carbonic anhydrase 3 (*Car3*), perilipin-1 (*Plin1*), 1-acyl-sn-glycerol-3-phosphate acyltransferase (*Agpat2*), carboxylesterase (*Ces1f*), platelet glycoprotein 4 (*Cd36*), and glycerol-3-phosphate dehydrogenase (*Gpd1*) (Figure 7A–G).

**Figure 8:**
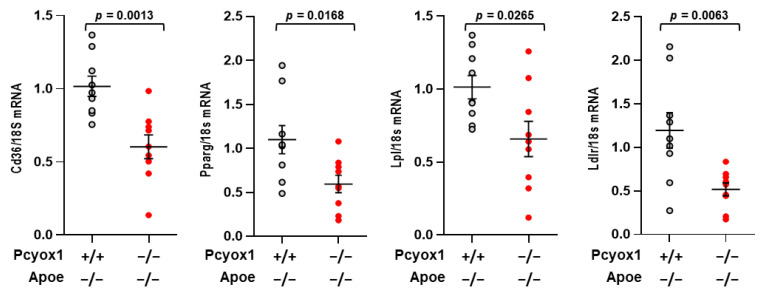
mRNA levels of *Cd36*, *Pparg*, Lpl and *Ldlr* in Pcyox1^−/−^/ApoE^−/−^ (*n* = 9) mice fed with HFD for 8 weeks compared with Pcyox1^+/+^/Apoe^−/−^ (*n* = 9); *18s* mRNA was used as a housekeeping gene. Data are presented as circle plot, with each circle representing an individual mouse and bars showing the mean value ± SEM, *n* = 9. Statistical significance calculated by Student’s *t* test.

We then evaluated gene expression in the VAT of mice fed an HFD for 8 weeks. As shown in Figure 8, *Pcyox1* deficiency was associated with a significant reduction in the expression of the genes involved in adipogenesis, such as *Cd36*, *Pparg*, *Lpl*, and *Ldlr* (Figure 8).

**Figure 9:**
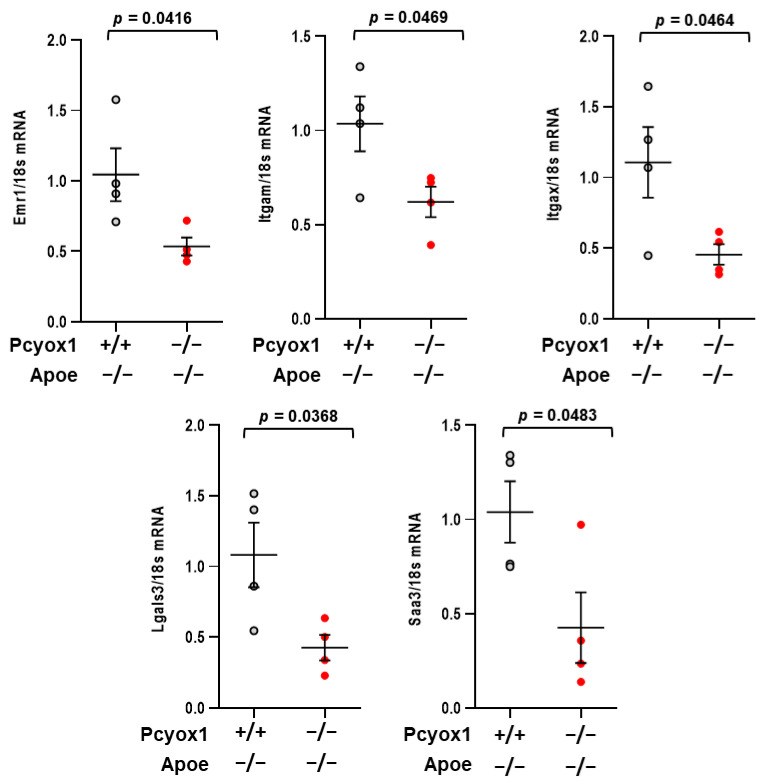
Changes in mRNA of *Emr1*, *Itgam* (CD11b), *Itgax* (CD11c), *Lgals3* (MAC2) and *Saa3* in Pcyox1^−/−^/Apoe^−/−^ mice (*n* = 4) fed with HFD for 8 weeks compared with Pcyox1^+/+^/Apoe^−/−^ (*n* = 4); *18s* mRNA was used as a housekeeping gene. Data are presented as circle plot, with each circle representing an individual mouse and bars showing the mean value ± SEM. Statistical significance calculated by Student’s *t* test.

To determine the effect of the ablation of *Pcyox1* on adipose tissue inflammation, we examined the expression of inflammatory markers serum amiloyd A3 (SAA3), MAC2, CD11b, CD11c, and EMR1. All of them were decreased in the VAT of Pcyox1^−/−^/Apoe^−/−^ mice versus Pcyox1^+/+^/Apoe^−/−^ mice after 8 weeks on HFD (Figure 9).

## 4. Discussion

New treatments that directly target adipose tissue accumulation may be possible if we understand all the factors necessary for normal adipogenesis. To the list of these factors, we add PCYOX1 as a novel regulator of adipogenesis. Our results demonstrate that PCYOX1 is expressed in adipose tissue, and that its deficiency results in reduced fat deposits. In agreement with these findings, in vitro experiments revealed that PCYOX1 is necessary for adipogenesis. PCYOX1 first emerged at the end of the 1990s as a lysosomal enzyme involved in the catabolism of prenylated proteins acting as a flavin adenine dinucleotide (FAD)-dependent thioether oxidase able to generate a stoichiometric amount of H_2_O_2_ [5,16]. Afterwards, thanks to the advent of proteomics, the knowledge of PCYOX1 was extended to the finding that this protein belongs to the proteome of lipoproteins, in which it contributes to their oxidative modifications [17]. Furthermore, *Pcyox1* silencing in vitro was found to affect the cellular proteome by influencing multiple functions related to inflammation, oxidative stress, and platelet adhesion [4,13]. We also showed that *Pcyox1* deficiency in Apoe^−/−^ mice on C57/BL6J background mice (B6.129P2-Apoetm1Unc/J) retards atheroprogression, is associated with decreased features of lesion vulnerability and lower levels of lipid peroxidation, and reduces plasma lipid levels and inflammation, thus highlighting for the first time the role of PCYOX1 in vivo. Indeed, the pioneering study of Beigneux et al. [18] showed the absence of any histologic abnormalities in a survey of >30 tissues from *Pcyox1*-deficient mice on a mixed C57BL/6-129/SvJae genetic background. The observation that *Pcyox1* deficiency in Apoe^−/−^ mice fed with HFD, a model of obesity-accelerated atherosclerosis accompanied by development of a metabolic syndrome phenotype [19], have less adipose tissue depots, led us to further investigate the role of PCYOX1 in adipogenesis.

The differentiation of preadipocytes into adipocytes is regulated by an elaborate network of transcription factors that coordinate the expression of hundreds of proteins responsible for establishing the mature fat-cell phenotype. At the center of this network are the two principal adipogenic factors, PPARγ and C/EBPα, which oversee the entire terminal differentiation process. PPARγ in particular is considered the master regulator of adipogenesis; without it, precursor cells are incapable of expressing any known aspect of the adipocyte phenotype [20]. 

We found that the knockdown of *Pcyox1* in 3T3-L1 preadipocytes renders them defective in inducing markers of terminal differentiation such as C/EBPα, PPARγ, and FABP4 and in accumulating fat, suggesting that PCYOX1 plays a pivotal role during the early events of the process. In this process, we ruled out the reciprocal regulation between PCYOX1 and C/EBPβ, suggesting that PCYOX1 acts independently from this crucial transcription factor. In an attempt to define the sequence of events leading to terminal adipogenesis, it was proposed that C/EBPβ and C/EBPδ simultaneously control the expression of both PPARγ and C/EBPα. Alternatively, some investigators have suggested that C/EBPβ induces C/EBPα and that, together, these factors regulate PPARγ expression [21]. The precise role of C/EBPβ and C/EBPδ in regulating this cascade of factors has been questioned, however, in knockout mice. Specifically, Tanaka et al. [22] demonstrated that adipocyte differentiation in vitro proceeds according to the proposed transcriptional regulatory cascade in which adipogenic transcription factors such as C/EBP family members and PPARs are activated sequentially. However, in vivo, C/EBPα and PPARγ can be induced without expression of C/EBPβ and C/EBPδ. These data suggest that there is some redundancy in the early steps of adipogenesis in vivo where alternative pathways operate to ensure the expression of PPARγ and C/EBPα. Over the last few years, many studies suggested that many additional transcription factors are potential components of this complex network of factors responsible for inducing adipogenic gene expression [23,24,25]. It is likely that additional factors of parallel pathways are induced early and converge on PPARγ at a stage downstream of C/EBPβ and C/EBPδ [21,25].

The evidence that PCYOX1 generates H_2_O_2_, which in turn leads to the oxidative modifications of lipoproteins [4], suggested that it might be involved in the synthesis of PPARγ ligands. Indeed, it has been reported that oxLDL induces PPARγ activation, and that 9-hydroxyoctadecadienoic acid (9-HODE), 13-hydroxyoctadecadienoic acid (13-HODE), and oxidized phospholipids, which are components of oxLDL, are involved in oxLDL-induced PPARγ activation [15]. However, our findings excluded that PCYOX1 might contribute to the adipogenic process by providing a ligand for PPARγ, as far as oxLDL is concerned. 

Furthermore, a peak of expression of PCYOX1 at later stages of 3T3-L1 differentiation highlights an additional role in terminal differentiation. Inactivation of *Pcyox1* blocked the expression of several mediators involved in adipogenesis, including, among others, Perilipin-1 (*Plin1*), 1-acyl-sn-glycerol-3-phosphate acyltransferase beta (*Agpat2*), Carboxylesterase 1 (*Ces1*), Hormone-sensitive lipase (*Lipe*), and Carbonic anhydrase 3 (*Car3*).

Importantly, Perilipin-1 is abundantly expressed in mature adipocytes, phosphorylated in a cAMP-dependent manner, and localized to lipid droplet surfaces during differentiation of 3T3-L1 adipocytes into lipid-accumulating mature adipocytes. *Plin1*-knockout (KO) mice exhibited striking phenotypes [26,27], being lean, with microscopically reduced lipid droplet sizes in adipose tissues, increased glucose tolerance and resistance to diet-induced obesity [28]. In addition, the high lipolytic activities in the WAT of *Plin1*-KO mice prevent the accumulation of triglycerides, suggesting that Perilipin-1 exerts essential roles in lipid droplet formation and triglyceride metabolism in vivo. 

Additionally, isoform 2 of the 1-acyl-sn-glycerol-3-phosphate acyltransferases (*Agpat2*) is a key enzyme involved in lipid synthesis, which is highly expressed in adipose tissue. It catalyses the acylation of lyso-phospatidic acid (LPA) to produce phosphatidic acid (PA), which will subsequently enter triglyceride or phospholipid synthesis. Cellular studies have supported that *Agpat2* is necessary for adipocyte differentiation, suggesting that the absence of WAT in *Agpat2* KO was the result of altered adipogenesis [29,30]. Furthermore, decreased levels of LPC in *Agpat2* KO could lead to reduced synthesis of LPA, which has been previously shown to be a physiological PPARγ ligand [31].

Recent advances in research have shown the relevance of carboxylesterases to metabolic diseases such as obesity and fatty liver disease [32]. Expression of *Ces1* was induced during 3T3-L1 adipocyte differentiation [33], and administration of Ces1 inhibitors to HFD fed mice or db/db mice protected from weight gain reduced plasma lipids, ameliorated liver steatosis, and improved glucose tolerance [34]. Furthermore, in the adipose tissue of obese and type 2 diabetic patients, the activity of Ces1 is elevated, which is consistent with other studies showing that Ces1 expression is higher in adipose tissue from obese patients compared to lean subjects [35,36]. 

Hormone-sensitive lipase (*Lipe*) is rate-limiting for diacylglycerol and cholesteryl ester hydrolysis in adipose tissue and essential for complete hormone-stimulated lipolysis [37]. Gene expression profiling in Lipe^−/−^ mice suggests that it is important for modulating adipogenesis and adipose metabolism. In vitro studies showed that Lipe increases during differentiation [38], likely providing ligands for the activation of PPARγ.

PCYOX1 also modulates *Car3,* which belongs to the family of carbonic anhydrases, enzymes involved in the promotion of fatty acid synthesis in adipocytes and the liver [39]. Car3 is highly abundant in tissues that can store lipids [40,41], and increases in rodents fed Western-type high-fat diets [42], becoming one of the most abundant transcripts in both human [43] and rodent [44] adipose tissues, accounting for up to 2% of the total mRNA. Moreover, Car3 constitutes the most abundant protein in mature adipocytes, comprising up to 24% of the total soluble protein fraction [40].

Overall, the modulation of a plethora of genes involved in adipogenesis supports the hypothesis that PCYOX1 not only acts at early stages of adipogenesis, but also may account for the persistent expression of genes relevant for terminal adipogenic differentiation. 

Importantly, PCYOX1 deficiency abrogated, in vitro and in vivo, the expression of CD36, a multifunctional immuno-metabolic receptor that is involved in many physiological and pathological processes [45]. Compared to wild-type mice, CD36 deficient mice with an HFD exhibited reduced adipose tissue inflammation, as evidenced by decreased pro-inflammatory cytokine levels in adipose tissue, and less macrophage and T-cell accumulation in adipose tissue [46,47].

Consistent with the role of PCYOX1 in the regulation of adipose tissue inflammation is the finding that SAA3, one of the members of the acute-phase proteins serum amyloid A family, is also downregulated in Pcyox1^−/−^/Apoe^−/−^ mice. Furthermore, SAA3 has been recently utilized for monitoring the adipose inflammatory state, possibly serving as an index of the number of infiltrated macrophages in adipose tissue [48]. In support of this hypothesis, we also found that the macrophage markers Mac2, Cd11b, Cd11c, and Emr1 were all decreased in the adipose tissue of *Pcyox1* deficient mice, thus substantiating the hypothesis of a crosstalk between adipocytes and infiltrated macrophages as an important pathological phenomenon leading to adipose tissue inflammation.

## 5. Conclusions

In summary, we report here that PCYOX1 is a novel regulator of adipogenesis, which acts upstream of known transcription factors and genes involved in lipogenesis, lipid droplet formation as well as lipid binding, and inflammation. These novel findings expand our knowledge on PCYOX1 biology and functions beyond its role in the metabolism of prenylated proteins [5,6,18,49]. Indeed, PCYOX1 is emerging as a multifunctional protein with a relevant role in atherogenesis by modulating lipid metabolism, inflammation and lipid peroxidation [4,17], in thrombosis by likely regulating circulating coagulant and inflammatory factors [13], and lastly, in adipogenesis. How PCYOX1 can modulate such different processes, which might not be restricted to its enzymatic pro-oxidant activity, is under investigation. Understanding the biology and mechanisms of all functions of this unique enzyme will help to provide additional therapeutic opportunities in addressing such conditions.

## Figures and Tables

**Table 1:** List of differentially expressed proteins in the proteome of differentiated 3T3-L1 cells after *Pcyox1* silencing with respect to control cells, treated with a negative construct.

Accession	Unique Peptides	*q* Value	Max Fold Change	Highest Mean Condition	Description
**Reduced in *Pcyox1* silenced cells**					
P54310	3	0.003	**7.07**	NEG	Hormone-sensitive lipase OS = Mus musculus GN = Lipe
Q9CQF9	2	0.001	**4.84**	NEG	Prenylcysteine oxidase OS = Mus musculus GN = Pcyox1
P16015	5	0.001	**4.73**	NEG	Carbonic anhydrase 3 OS = Mus musculus GN = Car3
P04117	5	0.003	**3.51**	NEG	Fatty acid-binding protein_ adipocyte OS = Mus musculus GN = Fabp4
Q8CGN5	5	0.001	**3.34**	NEG	Perilipin-1 OS = Mus musculus GN = Plin1
Q8K3K7	3	0.003	**2.91**	NEG	1-acyl-sn-glycerol-3-phosphate acyltransferase beta OS = Mus musculus GN = Agpat2
Q91WU0	2	0.006	**2.77**	NEG	Carboxylesterase 1F OS = Mus musculus GN = Ces1f
Q08857	2	0.012	**2.32**	NEG	Platelet glycoprotein 4 OS = Mus musculus GN = Cd36
P13707	9	0.006	**2.32**	NEG	Glycerol-3-phosphate dehydrogenase [NAD(+)]_ cytoplasmic OS = Mus musculus GN = Gpd1
P28271	3	0.004	**2.18**	NEG	Cytoplasmic aconitate hydratase OS = Mus musculus GN = Aco1
P16125	2	0.011	**2.03**	NEG	L-lactate dehydrogenase B chain OS = Mus musculus GN = Ldhb P
P24270	2	0.004	**1.92**	NEG	Catalase OS = Mus musculus GN = Cat
Q63918	4	0.001	**1.92**	NEG	Serum deprivation-response protein OS = Mus musculus GN = Sdpr
Q05920	18	0.001	**1.88**	NEG	Pyruvate carboxylase_ mitochondrial OS = Mus musculus GN = Pc
P31786	2	0.021	**1.87**	NEG	Acyl-CoA-binding protein OS = Mus musculus GN = Dbi
O88492	3	0.019	**1.86**	NEG	Perilipin-4 OS = Mus musculus GN = Plin4
P45952	3	0.006	**1.81**	NEG	Medium-chain specific acyl-CoA dehydrogenase_ mitochondrial OS = Mus musculus GN = Acadm
Q8BMS1	13	0.007	**1.80**	NEG	Trifunctional enzyme subunit alpha_ mitochondrial OS = Mus musculus GN = Hadha
P97807	6	0.004	**1.79**	NEG	Fumarate hydratase_ mitochondrial OS = Mus musculus GN = Fh
Q8BH95	3	0.037	**1.70**	NEG	Enoyl-CoA hydratase_ mitochondrial OS = Mus musculus GN = Echs1
Q9WTP7	2	0.007	**1.68**	NEG	GTP:AMP phosphotransferase AK3_ mitochondrial OS = Mus musculus GN = Ak3
P49817	2	0.016	**1.53**	NEG	Caveolin-1 OS = Mus musculus GN = Cav1
Q9CZU6	4	0.017	**1.51**	NEG	Citrate synthase_ mitochondrial OS = Mus musculus GN = Cs
P62897	2	0.005	**1.49**	NEG	Cytochrome c_ somatic OS = Mus musculus GN = Cycs
Q9EQ20	3	0.001	**1.48**	NEG	Methylmalonate-semialdehyde dehydrogenase [acylating]_ mitochondrial OS = Mus musculus GN = Aldh6a1
Q9JHI5	2	0.007	**1.48**	NEG	Isovaleryl-CoA dehydrogenase_ mitochondrial OS = Mus musculus GN = Ivd
Q9CZ13	3	0.009	**1.47**	NEG	Cytochrome b-c1 complex subunit 1_ mitochondrial OS = Mus musculus GN = Uqcrc1
O54724	6	0.001	**1.47**	NEG	Polymerase I and transcript release factor OS = Mus musculus GN = Ptrf
Q8BH64	8	0.003	**1.46**	NEG	EH domain-containing protein 2 OS = Mus musculus GN = Ehd2
P17751	9	0.001	**1.46**	NEG	Triosephosphate isomerase OS = Mus musculus GN = Tpi1
Q9D3D9	2	0.010	**1.46**	NEG	ATP synthase subunit delta_ mitochondrial OS = Mus musculus GN = Atp5d
Q8BMF4	5	0.011	**1.45**	NEG	Dihydrolipoyllysine-residue acetyltransferase component of pyruvate dehydrogenase complex_ mitochondrial OS = Mus musculus GN = Dlat
Q60597	4	0.022	**1.44**	NEG	2-oxoglutarate dehydrogenase_ mitochondrial OS = Mus musculus GN = Ogdh
Q9DB77	7	0.014	**1.43**	NEG	Cytochrome b-c1 complex subunit 2_ mitochondrial OS = Mus musculus GN = Uqcrc2
Q99L13	3	0.003	**1.42**	NEG	3-hydroxyisobutyrate dehydrogenase_ mitochondrial OS = Mus musculus GN = Hibadh
P08249	13	0.019	**1.42**	NEG	Malate dehydrogenase_ mitochondrial OS = Mus musculus GN = Mdh2
Q9DCW4	4	0.017	**1.42**	NEG	Electron transfer flavoprotein subunit beta OS = Mus musculus GN = Etfb
Q99LC5	9	0.011	**1.42**	NEG	Electron transfer flavoprotein subunit alpha_ mitochondrial OS = Mus musculus GN = Etfa
Q9CR62	2	0.003	**1.41**	NEG	Mitochondrial 2-oxoglutarate/malate carrier protein OS = Mus musculus GN = Slc25a11
P00405	4	0.046	**1.41**	NEG	Cytochrome c oxidase subunit 2 OS = Mus musculus GN = Mtco2
Q922Q1	2	0.034	**1.41**	NEG	Mitochondrial amidoxime reducing component 2 OS = Mus musculus GN = Marc2
P47738	8	0.016	**1.40**	NEG	Aldehyde dehydrogenase_ mitochondrial OS = Mus musculus GN = Aldh2
Q9D9V3	4	0.022	**1.40**	NEG	Ethylmalonyl-CoA decarboxylase OS = Mus musculus GN = Echdc1
**Increased in *Pcyox1* silenced cells**					
Q60847	4	0.001	**1.99**	shPCYOX1	Collagen alpha-1(XII) chain OS = Mus musculus GN = Col12a1
P37889	16	0.004	**1.42**	shPCYOX1	Fibulin-2 OS = Mus musculus GN = Fbln2

## Data Availability

Data collected in the study will be made available using the data repository Zenodo (https://zenodo.org accessed on 22 February 2023) with restricted access upon request to direzione.scientifica@ccfm.it. Proteomic data are available via ProeomeXchange with identifier PXD039943. Any remaining information can be obtained from the corresponding Author upon reasonable request.

## Supplementary Materials

The following supporting information can be downloaded at: https://www.mdpi.com/article/10.3390/antiox12030542/s1, Table S1: Primers used for qRT-PCR; Table S2: Primers purchased from Qiagen; Table S3: List of eEnriched GO terms in the biological process category considering all the proteins modulated by Pcyox1 silencing.
