# Supplementary material for: Prenylcysteine Oxidase 1 Is a Key Regulator of Adipogenesis
Source: Antioxidants (Basel). 2023 Feb 21;12(3):542. doi: 10.3390/antiox12030542 (PMC10045348; doi:10.3390/antiox12030542)
Supplement: Supplementary file 1 [file antioxidants-12-00542-s001.zip › Table S1.pdf]

Table S1. Primers used for qRT-PCR

Gene	Sequence Fwd	Sequence Rev
18s	5' – GTA ACC CGT TGA ACC CCA TT – 3'	5' – CCA TCC AAT CGG TAG TAG CG – 3'
Gapdh	5' – CGT GCC GCC TGG AGA AAC C – 3'	5' – TGG AAG AGT GGG AGT TGC TGT TG – 3'
Lpl	5' – TTG CCC TAA GGA CCC CTG AA – 3'	5' – ACA GAG TCT GCT AAT CCA GGA AT – 3'
Cd36	5' – ATG GGC TGT GAT CGG AAC TG – 3'	5' – AGC CAG GAC TGC ACC AAT AAC – 3'
Ldlr	5' – TGA CTC AGA CGA ACA AGG CTG – 3'	5' – ATC TAG GCA ATC TCG GTC TCC – 3'
