# Supplementary material for: Prenylcysteine Oxidase 1 Is a Key Regulator of Adipogenesis
Source: Antioxidants (Basel). 2023 Feb 21;12(3):542. doi: 10.3390/antiox12030542 (PMC10045348; doi:10.3390/antiox12030542)
Supplement: Supplementary file 1 [file antioxidants-12-00542-s001.zip › Table S2.pdf]

Table S2. Primers purchased from Qiagen

Primers	Reference
Mm_Pcyox1_1_SG QuantiTect Primer Assay	QT00115150
Mm_Pparg_1_SG QuantiTect Primer Assay	QT00100296
Mm_Fabp4_1_SG QuantiTect Primer Assay	QT00091532
Mm_Cebpb_1_SG QuantiTect Primer Assay	QT00320313
Mm_Cebpa_1_SG QuantiTect Primer Assay	QT00311731
Mm_Lipe_1_SG QuantiTect Primer Assay	QT00169057
Mm_Car3_1_SG QuantiTect Primer Assay	QT00101038
Mm_Plin1_1_SG QuantiTect Primer Assay	QT00150360
Mm_Agpat2_1_SG QuantiTect Primer Assay	QT00104888
Mm_Ces1f_2_SG QuantiTect Primer Assay	QT01167971
Mm_Gpd1_1_SG QuantiTect Primer Assay	QT00154777
Mm_Emr1_1_SG QuantiTect Primer Assay	QT00099617
Mm_Itgam_1_SG QuantiTect Primer Assay	QT00156471
Mm_Itgax_1_SG QuantiTect Primer Assay	QT00113715
Mm_Lgals3_1_SG QuantiTect Primer Assay	QT00152558
Mm_Saa3_1_SG QuantiTect Primer Assay	QT00249823
